# Mechanisms and Functions of γδ T Cells in Tumor Cell Recognition

**DOI:** 10.3390/curroncol32060329

**Published:** 2025-06-03

**Authors:** Jing Tang, Chen Wu, Jintong Na, Yamin Deng, Simin Qin, Liping Zhong, Yongxiang Zhao

**Affiliations:** 1 State Key Laboratory of Targeting Oncology, National Center for International Research of Bio-Targeting Therangstics, Guangxi Key Laboratory of Bio-Targeting Therangstics, Collaborative Innovation Center for Targeting Tumor Diagnosis and Therapy, Guangxi Talent Highland of Major New Drugs Innovation and Development, Guangxi Medical University, Nanning 530021, China; tang_jing@sr.gxmu.edu.cn (J.T.); 202120287@sr.gxmu.edu.cn (C.W.); najintong@sr.gxmu.edu.cn (J.N.); yamin_deng@sr.gxmu.edu.cn (Y.D.); 202221577@sr.gxmu.edu.cn (S.Q.); 2 Pharmaceutical College, Guangxi Medical University, Nanning 530021, China

**Keywords:** γδ T cells, phosphoantigen, BTN3A1, adoptive therapy, tumor cell

## Abstract

γδ T cells are among the first line of defense in the immune system, playing a crucial role in bridging innate and adaptive immunity. Although γδ T cells are crucial for tumor immune surveillance, the complete mechanism by which γδ T cell receptors identify molecular targets in target cells remains unknown. Target cells can produce phosphoantigens (PAgs) via the mevalonate pathway or the methylerythritol phosphate pathway. The BTN3A1–BTN2A1 complex undergoes conformational changes in its extracellular domains upon binding to PAgs, leading to Vγ9Vδ2 T cell recognition. However, the structural basis of how Vγ9Vδ2 T cells recognize changes in this complex remains elusive. This review provides a detailed overview of the historical progress and recent discoveries regarding how Vγ9Vδ2 T cells recognize and target tumor cells. We also discuss the potential of γδ T cells immunotherapy and their role as antitumor agents.

## 1. Introduction

T cells play a pivotal role in shaping the tumor microenvironment (TME). Historically, research and therapeutic strategies have focused on the T cells expressing αβ T-cell receptors (TCRs), with less attention paid to γδ T cells. However, in addition to αβ T cells, γδ T cells—a non-conventional subset characterized by γδ TCRs—have shown remarkable antitumor potential and are gaining attention in immunotherapy [1,2]. While αβ T cells primarily recognize the antigens presented by major histocompatibility complex (MHC) molecules [3], Vγ9Vδ2 T cells generally identify tumors without MHC, often via butyrophilin (BTN) complexes on tumor cell surfaces [4].

**Figure 1:**
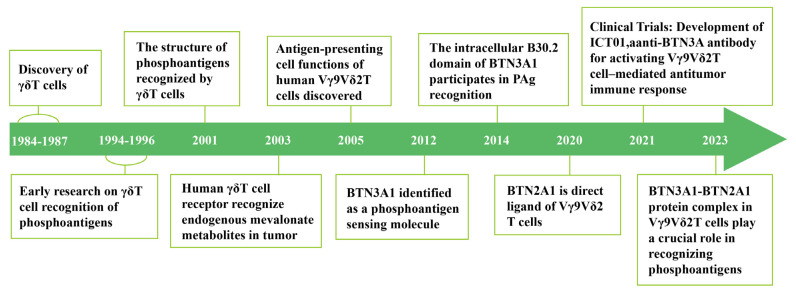
Chronology of advancements in the investigation of γδ T cell’s roles in tumors and their phosphoantigen recognition capabilities. 1984–1987 (Discovery of γδT cells [7]); 1991–1994 (Early research on γδT cell recognition of phosphoantigens [9,10]); 2001 (The structure of phosphoantigens recognized by γδT cells [11]); 2003 (Human γδT cell receptor recognize endogenous mevalonate metabolites in tumor [12]); 2005 (Antigen-presentingcell functions ofhuman Vγ9Vδ2T cells discovered [13]); 2012 (BTN3A1 identified as a phosphoantigensensing molecule [14]); 2014 (The intracellular B30.2 domain of BTN3A1 participates in PAg recognition [15]); 2020 (BTN2A1 is direct ligand of Vγ9Vδ2 T cells [16]); 2021 (Clinical Trials: Development of ICT01,a anti-BTN3A antibody for activating Vγ9Vδ2T cell–mediated antitumor immune response [8]); 2023 (BTN3A1-BTN2A1 protein complex in Vγ9Vδ2T cells play a crucial role inrecognizing phosphoantigens [17]).

Recent preclinical studies using human and mouse models have reported significant differences in TCR repertoires between humans and mice [5]. Human γδ T cells can be categorized into three main subgroups, Vδ1+, Vδ2+, and Vδ3+ T cells, primarily based on the surface antigen Vδ chain (Table 1) [6]. Despite species-specific TCR repertoire differences, γδ T cells in humans and mice exhibit similar antitumor functions [7], allowing for rapid research on γδ T cells and providing a more comprehensive understanding of their immunological functions. Numerous clinical trials have attempted to harness their potential for immunotherapy [8]. Therefore, the accumulation of knowledge on the γδ T cell lineage has experienced exponential growth in recent years. This review summarizes recent findings on the γδ T cell’s ability to recognize phosphoantigen (PAg) sources and structures; the mechanisms by which they recognize these antigens; their antitumor effects, particularly within the TME; and the promising potential of γδ T-cell adoptive therapy (Figure 1).

**Table 1:** Classification of γδ T cells.

**γδ T-Cell Subset**	**Paired Vγ Gene Usage**	**Distribution**	**Features**	**Tumor-Related Functions**	**References**
Vδ1+T cells	Vγ2/3/4/5/8/9	PB, skin, gut, Spleen, liver	Express NK cell receptors, Toll-like receptors, co-stimulatory factors; exhibit cytotoxicity against tumor cells via IFN-γ, and IL-10; low levels of IL-4, perforin, and granzyme	1. Promote tumor growth by secreting cytokines like IL-17 that induce vascular endothelial growth factor (VEGF) secretion from tumor cells 2. Antitumor effect: In some hematological malignancies (such as leukemia), the clonal expansion and cytotoxicity of adult Vδ1 cells may enhance the tumor-killing ability, which is associated with a better prognosis	[18,19,20,21,22,23]
Vδ2+T cells	Vγ9	PB	Mainly Vγ9Vδ2 T cells responding to phosphorylated non-peptide “PAgs”; categorized into subgroups based on CD27 and CD45RA expression; naive and central memory cells respond to isopentenyl pyrophosphate (IPP), effector memory cells produce high IFN-γ, and terminally differentiated cells secrete perforin and granzyme	Effector memory Vδ2+ T cells have strong antitumor capacity, while terminally differentiated cells exert cytotoxic effects; activated Vδ2+ T cells can serve as antigen-presenting cells (APCs)	[24,25,26,27]
Vδ3+T cells	Vγ2/3	PB, liver	Express CD56, CD161, NKG2D; enhance CD1d recognition and act on CD1d target cells expressing CD107a	Limited investigations: functional role in tumor-related studies is not well defined	[28,29]
Vδ5+T cells	Vγ4	PB	EPCR		[16]
**Functional Subsets**		**Source**	**Secreted Cytokines**	**Function**	**References**
IFN-γ+ γδ T cell		thymus origin	IFN-γ	Functionally diverse: autoimmune diseases and tumor surveillance	[30]
IL-17+ γδ T cell		Vδ1γδ T-cell subpopulation of thymus origin	IL-17;	Rapid induction of IL-8-mediated migration and phagocytosis of neutrophils	[31]
γδ Treg		Vδ1γδ T-cell subpopulation	IFN-γ; GM-CSF	Inhibitory effect on the proliferation of autologous innate CD4+T cells	[32]
γδ T-APC				Initiated a specific immune response	[13]
TIGIT + γδ T		Vδ1γδ T cells		Dysfunctional effector state	[33]

PB, Peripheral Blood; IL-17, Interleukin 17; ULBPs, UL16 binding proteins; B7-H6, B7 homolog 6; IFN-γ, Interferon-γ; BTN3A1, butyrophilin subfamily 3 member A1; EPCR, Endothelial Protein C Receptor; GM-CSF, Granulocyte-Macrophage Colony-Stimulating Factor.

## 2. Mechanisms Underlying Tumor Cell Recognition and the Stimulation of γδ T Cells

Tumors arise under immune surveillance, with T cells recognizing specific antigenic markers expressed by tumor cells, leading to their eradication [34]. T-cell immune recognition can be broadly categorized into direct recognition mediated by MHC molecules and indirect recognition mediated by APCs, which process and present antigens via MHC molecules. The immune recognition mechanism of αβ T cells involves the specific recognition of the peptide antigens displayed on the exterior of APCs, binding with MHC molecules into complexes (polypeptide antigens can be anchored to the extracellular segment of MHC), and initiating the activation of αβ T cells; however, the recognition of antigenic peptides by αβ T cells is limited by the type of MHC molecules [35]. Unlike αβ T cells, past studies on the molecular mechanisms of γδ T cell recognition have revealed their unusual peptide-independent, non-MHC-restricted recognition. One remarkable characteristic of Vδ2+ T cells is their ability to serve as APCs. However, since the discovery of γδ T cells in the mid-1980s, research on their recognition of protein antigens appears to suggest direct recognition without processing and presentation. Studies show that γδ TCR does not recognize small non-peptide, phosphate-containing molecules but detects their upregulation through interactions with BTN complexes [36].

### 2.1. Early Studies on γδ T-Cell Activation: The Initial Discovery of PAgs

TUBag4 is a key non-peptide ligand promoting Vγ9Vδ2 T cell expansion. Initial investigations on γδ T cells documented an increase in Vγ9Vδ2 T cells in lesions infected with various bacteria and parasites, as well as in peripheral blood. In vitro investigations demonstrated that Vγ9Vδ2 T cells could respond effectively when stimulated with mycobacteria extracts [37]. In 1994, Constant and colleagues isolated four distinct water-soluble compounds (TUBag1–4) from the Mycobacterium tuberculosis H37RV strain. TUBag4 was identified as 5′-triphosphoryl thymidine, with the γ-phosphate group replaced by a low-molecular-weight moiety. TUBag4 stimulates the expansion of peripheral blood Vγ9Vδ2 T cells, supporting the hypothesis that γδ T-cell activation is related to non-peptide ligands [9].

IPP and Dimethylallyl pyrophosphate (DMAPP) play crucial roles in allowing Vγ9Vδ2 T cells to identify the stress signals specifically expressed by tumor cells, which, when produced by normal cells, are usually too weak to induce any Vγ9Vδ2 T cell reactivity. Initially, bacteria and parasites were demonstrated to generate potent Vγ9Vδ2 TCR PAg excitation agents. Subsequently, it was found that Vγ9Vδ2 T cells could be stimulated by weaker agonists as well, including IPP and DMAPP, which serve as natural intermediates in the mevalonate pathway (MVP) of isopentenyl diphosphate and perform sterol synthesis within eukaryotic cells [38]. IPP in Mycobacterium smegmatis has been identified as an inaugural natural agonist of Vγ9Vδ2 T cells. Various pathogens generate IPP, its isomer DMAPP, and (E)-4-Hydroxy-3-methyl-but-2-enyl pyrophosphate (HMBPP), an array of compounds commonly referred to as “PAgs”, via the methylerythritol phosphate pathway [39]. In 2001, Morita et al. tested the biological activity of synthetic analogs to determine the structural characteristics of isopentenyl pyrophosphate ester antigenicity [10]. PAgs with different structures also differ in their capacity to activate Vγ9Vδ2 T cells (Table 2). In 2023, Zhang et al. discovered that the unique hydroxyl head of HMBPP pyrophosphate forms two hydrogen bonds with amino acids in the intracellular segment of the butyrophilin subfamily 3 member A1 (BTN3A1) (explained in detail in the next section). This hydrogen bonding is not observed with the endogenous antigens DMAPP and IPP [17]. This explains why pathogen-generated HMBPP can activate Vγ9Vδ2 T cells better than the endogenous DMAPP and IPP.

Although IPP may stimulate refined Vγ9Vδ2 T cells, preliminary investigations revealed that cell-to-cell contact between autologous and non-autologous T cells is the minimum requirement [40]. This indicates that the direct stimulation of Vγ9Vδ2 T cells involves the participation of co-stimulatory molecules or adhesion molecules. The reactivity of Vγ9Vδ2 T cells to tumor surfaces corroborates this hypothesis [41]. Furthermore, Vγ9Vδ2 T cells cross-link photoreactive PAg on their surface and show stimulation without antigen-presenting molecules [42].

**Table 2:** Bioactivities of different PAgs.

Name	Specific Source	Biological Activity	Research Progress	References
TUBag4	Mycobacterium tuberculosis H37RV strain	Stimulates Vγ9Vδ2 T cell expansion supports the hypothesis of γδ T cell recognition of non-peptide ligands	First isolated from M. tuberculosis, confirming non-peptide ligands can activate Vγ9Vδ2 T cells.	[9]
IPP	Tumor cells, bacteria (e.g., Mycobacterium smegmatis), eukaryotic mevalonate pathway	Weak agonist requires higher concentrations to activate Vγ9Vδ2 T cells	Natural intermediate of MVP in eukaryotic cells, increased expression in tumor cells.	[12]
DMAPP	Tumor cells, bacteria, eukaryotic mevalonate pathway	Weak agonist, similar to IPP, requires higher concentrations to activate Vγ9Vδ2 T cells	Like IPP, an intermediate of MVP, increased expression in tumor cells.	[12]
HMBPP	Bacteria (e.g., *E. coli*, *M. tuberculosis*), parasites (e.g., Plasmodium) via MEP pathway	Strongest natural agonist, activates Vγ9Vδ2 T cells at very low concentrations	In 2023, its hydroxyl group was found to form hydrogen bonds with BTN3A1, explaining its high potency.	[17]
BrHPP	Synthetic compound (modified from natural phosphoantigen structures)	Highly efficient synthetic activator, activity close to HMBPP	Widely used in clinical research as a substitute for HMBPP.	[11]
Zoledronic Acid (ZOL)	Synthetic amino bisphosphonate (originally developed for osteoporosis treatment)	Indirectly activates Vγ9Vδ2 T cells by inhibiting the MVP pathway and increasing IPP levels	Used in immunotherapy, confirming its immunomodulatory effects.	[43]
Pamidronate	Synthetic amino bisphosphonate	Indirectly activates Vγ9Vδ2 T cells by inhibiting the MVP pathway and increasing IPP levels	Similar to ZOL, it is used in cancer treatment research.	[44]

### 2.2. How γδ T Cells Detect PAg: BTN3A Family

The human BTN gene superfamily is a part of the B7 protein superfamily [45]. BTN genes comprise at least 10 subgroups in mice and have been identified in humans, with 13 members on chromosome 6p. The BTN molecules BTN3A1, BTN3A2, and BTN3A3 form the BTN3A subfamily [46]. A distinctive feature of BTN3A1 is a curled α-helix structural domain at the N-terminus of the B30.2 domain, which is associated with the transmembrane domain [47]. BTN3A1 and -A3 include the B30.2 domain, whereas BTN3A2 does not have this domain, and the B30.2 domain present in BTN3A3 cannot interact with PAgs. Only the B30.2 intracellular domain of BTN3A1 is capable of directly binding with PAgs through a highly positively charged surface pocket. Therefore, a single amino acid substitution at position 351 in the B30.2 domain of BTN3A3, where histidine (present in BTN3A1) is replaced by arginine (in BTN3A3), prevents the binding of PAgs to this surface pocket [15].

Vγ9Vδ2 T cells recognize PAgs in a unique manner that is distinct from the recognition of antigens by other immune cells. In humans and primates, Vγ9Vδ2 T cells exhibit rapid activation and proliferation in response to the PAgs produced by bacteria and tumors, thereby rapidly exerting cytotoxicity. However, the precise molecular basis of the Vγ9Vδ2 T cell recognition of PAgs remains unclear. Initially, it was speculated that extracellular PAgs are present in Vγ9Vδ2 T cells (similar to MHC molecules). However, in 1992, Correa and colleagues refuted this model by demonstrating that Vγ9Vδ2 T cells exhibited no apparent defects in cell structure, marker expression, overall function, or functional activity in β2-microglobulin mutant mice [48]. Additionally, cells lacking β2-microglobulin readily stimulate Vγ9Vδ2 T cells. Therefore, it was previously established that MHC and MHC-like molecules are not involved in PAg-dependent activation and that inhibitory antibodies against these molecules do not significantly affect Vγ9Vδ2 T-cell activation [49]. This finding clarifies that Vγ9Vδ2 T cells recognize PAgs through a mechanism independent of the classical antigen presentation pathways involving MHC molecules.

The activation of Vγ9Vδ2 T cells by BTN3A subtypes suggests that these molecules play a crucial role in recognizing and responding to PAgs. The specific involvement of BTN3A1 underscores its critical importance in the recognition and activation of Vγ9Vδ2 T cells by PAgs. A pioneering discovery in the identification of key components recognizing PAg was made by Harly et al. in 2012 [14]. They found that the anti-CD277 antibody 20.1 can replicate the PAg-induced stimulation of Vγ9Vδ2 T cells. This antibody was initially developed to explore the role and distribution of BTN proteins, and this serendipitous discovery has become crucial for researching Vγ9Vδ2 T-cell activation. This finding highlights the indispensable role of BTN3A1 in activating Vγ9Vδ2 T cells. The role of BTN3A1 in the PAg-triggered response of Vγ9Vδ2 T cells has been demonstrated, revealing that BTN3A1 is not a critical costimulatory or adhesion molecule but rather an essential protein for the Vγ9Vδ2 T cell recognition of PAgs. Subsequent studies have provided insights into this phenomenon. In 2014, the Sandstrom group clarified the structural, biophysical, and functional methods by which the intracellular B30.2 domain of BTN3A1 detects heightened intracellular PAg concentrations (such as those accumulated during tumor development) through a highly positively charged surface pocket, with all known PAgs containing negatively charged phosphates. The positively charged surface pocket directly binds to PAgs, inducing the fixation of the extracellular domain surface of BTN3A1 [15].

The requirement for a heterodimer configuration indicates that the structural integrity of the B30.2 domain is essential for effective Vγ9Vδ2 T-cell activation by PAgs. In a subsequent study, the Salim group in 2017 found that the direct bonding of PAg with the B30.2 domain induces a specific conformational change and extensive chemical shifts in the B30.2 domain (from fluctuation at the binding site to the distal portion of the domain), confirming that PAg combines with the B30.2 domain rather than the distal IgV domain [50]. Additionally, this finding reveals that this specific conformational change is only induced by PAg and not by other non-antigenic molecules, suggesting that the capability of PAg to provoke distinct conformational changes may underlie the activation of Vγ9Vδ2 T cell-induced specific recognition of target cells. Recently, in 2019, Yunyun Yang et al. used crystallography and chemical probe methods to demonstrate that HMBPP combines with the B30.2 domain, causing a conformational transition of histidine 351 from a β to an α isomer, leading to the induction of the B30.2 domain (a dimerization of the domain around the symmetric interface of the curled N-terminal α-helix, moving toward the asymmetric dimer interface). This resulted in an increase in the dimer interface area, affecting the fluctuation of each monomer, which propagates to the juxtamembrane (JM) region [51]. Thus, it was confirmed that Vγ9Vδ2 T cell stimulation requires a heterodimer configuration of the B30.2 domain.

The control of BTN3A oligomerization by the JM region highlights the intricate molecular interactions necessary for the functional assembly and signaling of these molecules during PAg recognition. The speculation regarding the roles of the three BTN3A molecules in recognizing PAg and activating Vγ9Vδ2 T cells has persisted, making it essential to elucidate the collaboration and allocation of tasks among different BTN3A proteins. In 2023, the Karunakaran group observed that the JM region of BTN3A1 exhibited strong positive charges. In contrast, there were two glutamic acid residues with a negative charge at the same position in BTN3A2 and BTN3A3. This difference makes the dimerization of BTN3A1 unstable due to electrostatic helical interactions, whereas the heterodimerization of BTN3A2 and BTN3A3 is more favorable [52]. Additionally, the homotypic or heterotypic oligomerization of BTN3A molecules is controlled by the JM region of BTN3A. Research has shown that when PAg binds to BTN3A, the BTN3A molecule forms a complex with a pair of BTN3A molecules through the JM region and transports them to the cell membrane [53]. In BTN3A heterooligomers, PAg binds to BTN3A1-B30.2 and associates with the BTN2A1-B30.2 domain, facilitating Vγ9Vδ2 T-cell activation. The complete IgV domain of BTN3A molecules is pivotal for PAg-mediated recognition by Vγ9Vδ2 TCR, another important finding published by the Karunakaran group [54]. Each paired BTN3A chain in the dimer must have a complete IgV domain, and the absence of the membrane-distal IgV domain inhibits BTN3A complex transport to the cell membrane and PAg stimulation. Still, these functions can be rescued through the cooperative action of paired BTN3A molecules [55,56]. The necessity of a complete IgV domain for proper functioning emphasizes its critical role in the trafficking and activation of BTN3A complexes in response to PAgs.

### 2.3. Various Factors Induce Changes in Cell Membrane Fluidity

**Figure 2:**
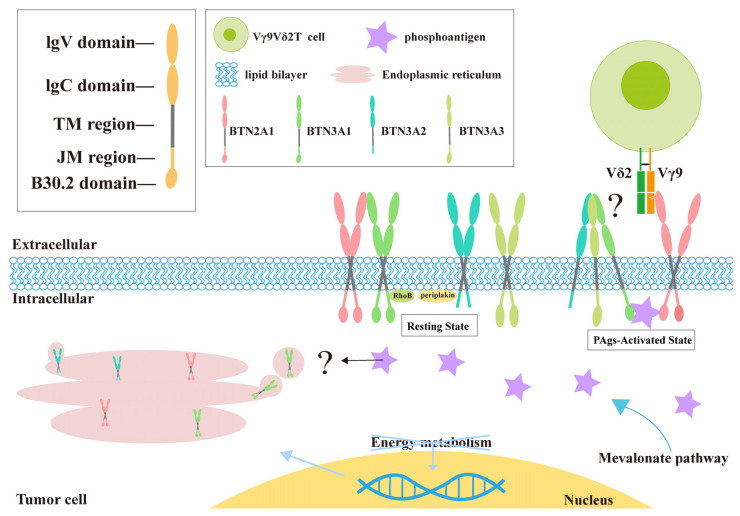
The process by which Vγ9Vδ2 T cells identify tumor cells is as follows: Molecules of the BTN family, belonging to the immunoglobulin superfamily, typically comprise two extracellular immunoglobulin-like domains (one membrane-distal IgV and one membrane-proximal IgC), a single transmembrane region, and a B30.2 domain situated in the cytoplasm (with variations in the intracellular domains among family members) and exhibit structural similarity to the B7 family in the extracellular domain. The abnormal energy metabolism of tumor cells leads to the increased expression of BTN family genes, resulting in the increased production of BTN molecules. PAg accumulates in tumor cells and binds with the B30.2 domain to form a complex with BTN2A1 and BTN3A2. This intracellular binding induces changes in the external domain of the BTN protein molecules, transmitting tumor cell signals from inside to outside the cell, where they can then be recognized by Vγ9Vδ2 T cells. ? means is still unclear whether the accumulation of phosphate antigens will lead to the accumulation of BTN proteins.

Initially, PAgs were hypothesized to present extracellularly rather than binding within tumor cells to activate Vγ9Vδ2 T cells. However, subsequent research refuted this model. It was found that Vγ9Vδ2 T cells cannot directly recognize PAg but require an intracellular BTN3A1 domain to differentiate between PAgs and non-antigenic small molecules. BTN3A1 undergoes conformational changes upon PAg binding, sensed by Vγ9Vδ2 TCR, subsequently activating Vγ9Vδ2 T cells [51]. This intracellular binding-induced alteration in the extracellular conformation enables Vγ9Vδ2 T cells to identify targets, a process known as intracellular-to-extracellular signaling (Figure 2).

The internal sensing of PAg must be converted to external signals to be recognized by Vγ9Vδ2 TCR. Vγ9Vδ2 T cell stimulation entails detecting metabolic changes in tumor cells, where the intracellular accumulation of PAg in tumor cells is closely associated with reduced membrane fluidity induced by the B30.2 domain, possibly due to membrane reorganization. The specific interaction between BTN3A1 and the cytoskeletal linker protein, and possibly other members of the protein group, is crucial for anchoring or stabilizing BTN3A signaling complexes within the cell membrane [57]. The group of Zsolt Sebestyen in 2016 found that RhoB modulates the capacity of tumor cells to stimulate Vγ9Vδ2 T cells by coordinating BTN3A1 within the cell membrane [58]. MVP dysfunction in tumor cells causes PAg accumulation, resulting in the activation of RhoB and its repositioning from the nucleus to the vicinity of BTN3A1. This process demands GTPase activity, regulating BTN3A1 membrane fluidity and BTN3A1 dimer membrane reorganization through cytoskeletal rearrangement, inducing conformational changes in the extracellular domains of BTN3A1 (independent of RhoB activity), facilitating binding to the TCR, and ultimately activating Vγ9Vδ2 T cells [59]. The expulsion of RhoB is a hallmark of tumor cells targeted by Vγ9Vδ2 T cells.

### 2.4. BTN2A1 Serves as a Direct Ligand for Vγ9Vδ2 T Cells

The second major perspective for identifying the key components recognizing PAg is demonstrating that BTN2A1 is a crucial ligand for binding to the Vγ9+ TCR γ chain. In 2020, Rigau M and colleagues gained clearer insights into the molecular basis of PAg recognition [60]. The surface binding of BTN2A1 and BTN3A1 on target cells is required to recognize these PAgs. The extracellular and intracellular domains of BTN2A1 and BTN3A1 are interconnected. BTN2A1 is associated with the Vγ9+ region. However, a second ligand, currently unidentified, binds to the Vδ2+ domain and γ-chain region on the opposite side of the TCR. Genome-wide screening revealed that BTN2A1 was distinct from BTN3A1. Without BTN2A1, Vγ9Vδ2 T cells failed to respond to small-molecule PAg [61]. Therefore, BTN2A1 serves as an immediate ligand for Vγ9Vδ2 TCR and is necessary for the Vγ9Vδ2 T cell-induced destruction of tumor cells [62,63].

Early studies suggested that BTN3A1 monomers alone are unlikely to incite PAg-induced extracellular conformational changes. Zhang et al. revealed that multiple PAgs act as “molecular glue” to facilitate the intracellular hetero-oligomerization of BTN3A1 and BTN2A1 [17]. BTN3A1 has an “immunocompanion”—BTN2A1—and both proteins act synergistically, participating in the capture of PAgs, thus endowing Vγ9Vδ2 T cells with “superior” immune surveillance capabilities. Even if only a small amount of PAg is present in tumor cells and pathogens, it can be efficiently “locked”. X-ray crystallography revealed that the combination of BTN3A1 and PAgs formed a complex directly bound to BTN2A1, and several PAgs were located in the center of the interface and bonded with different affinities. After cross-linking BTN3A1 and BTN2A1 intracellular domains, the outward conduction of the PAg-mediated BTN complex wave induces epitope exposure in the extracellular domain, effectively binding to the TCR and activating Vγ9Vδ2 T cells. BTN3A1 and BTN2A1 often accompany each other; their external sites are bound together, and their internal sites are separated. When PAg acts as a “glue” to bind the two intracellular sites, the previously adjacent extracellular binding sites are separated, triggering the extracellular conformational changes detected by Vγ9Vδ2 TCR [64].

Although understanding the association between Vγ9Vδ2 T-cell activation and PAgs is sufficient, it is more important to understand the regulatory mechanisms controlling the BTN complex. In 2023, the Mamedov group identified a new pathway for the Vγ9Vδ2 T cell recognition of tumor cells through CRISPR screening, involving the BTN2A1-3A1-3A2 heteromer acting as a “beacon” [65]. This pathway is regulated by multilevel processes, with tumor cell energy metabolism activating ATP-activated protein kinase [66,67], leading to the upregulation of the BTN2A1–BTN3A1–BTN3A2 complex and the enhanced binding of PAg, thereby enabling Vγ9Vδ2 T cells to recognize and exhibit cytotoxicity toward tumor cells [68].

## 3. The Two-Fold Function of γδ T Cells in the TME

### 3.1. The Direct Antitumor Effect of γδ T Cells

**Figure 3:**
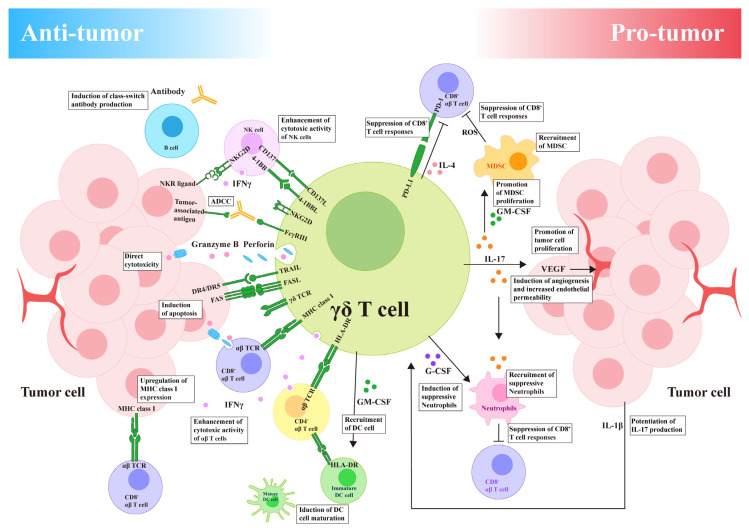
The dual role of γδ T cells in the tumor microenvironment (TME). Antitumor: γδ T cells can produce IFN-γ, which activates other immune cells. γδ T cells can present antigens to αβ T cells. γδ T cells exhibit inherent cytotoxic capabilities. Pro-tumor: γδ T cells secrete immunosuppressive cytokines such as interleukin (IL)-17A. IL-17A attracts immunosuppressive cells like MDSCs to the TME. γδ T cells contribute to the accumulation of MDSCs and neutrophils in the TME by producing chemokines. MDSCs and neutrophils can inhibit the function of cytotoxic T cells.

The biological functions of γδ T cells largely depend on cytokine secretion and cytotoxicity against target cells [69]. Upon activation, γδ T cells can eradicate tumor cells via various mechanisms (Figure 3). The antitumor biological functions of γδ T cells can be broadly classified into five pathways:

Direct killing of tumor cells: Upon activation, γδ T cells can directly induce tumor cell lysis by liberating perforin and granzyme B [70].

Cytokine-mediated cytotoxicity [71]: (1) Interferon-γ (IFN-γ) and TNF-α induce effects resembling those of Th1. IFN-γ, the primary cytokine, enhances cytotoxicity against tumor cells and the TME [72]. (2) Interleukin (IL)-4 and IL-10 induce effects resembling those of Th2, inhibiting the expansion of CD8+ T cells [73].

ADCC: γδ T cells express FcγRIII (CD16) receptors on their surface, enabling them to attach to the Fc portion of immunoglobulin G and facilitating the cytotoxic effect of antibodies against tumor cells. Additionally, they enhance cytotoxicity through IL-2 secretion [74].

Receptor–ligand binding pathways: (1) The Fas/FasL pathway: activated γδ T cells increase FasL expression, leading to tumor cell apoptosis through caspase cascade activation. (2) The TRAIL pathway: when death receptors (DR4 and DR5) bind to TRAIL, the intracellular death domains of these receptors initiate cytotoxic signals [75].

Recognition of tumor antigens for cytotoxicity: Other receptors such as NKG2D, NKp30, NKp44, and DNAM-1 (CD226), similar to natural killer (NK) cells, facilitate the recognition and elimination of tumor cells [76]. γδ T cells can limitlessly identify tumor cells via receptors such as NKG2D, even without human leukocyte or tumor antigens [77]. Furthermore, γδ T cells exhibit a higher infiltration capacity and functionality in the hypoxic TME.

### 3.2. Coordination of Additional Cells by γδ T Cells in Antitumor Activity

Activated γδ T cells can enhance tumor cell death by coordinating with additional immune cells.

αβ T cells: Studies have shown that γδ T cells can amplify the release of IFN-γ by αβ T cells (in B16 melanoma), presenting antigens to CD4+ T cells and cross-presenting antigens with CD4+ T cells to CD8+ T cells, thereby stimulating targeted immune reactions and exerting antitumor effects [78]. Moreover, γδ T cells upregulate the presentation of MHC-I and MHC-II molecules, promoting antigen presentation on tumor cells and improving the recognition of malignant cells by CD8+ T cells. Additionally, γδ T cells provide co-stimulatory cues that spur the expansion and maturation of naive αβ T cells.

B cells: γδ T cells can serve as APCs, inducing antibody production by B cells, enhancing humoral immune responses, regulating TNF-α secretion, and initiating specific immune responses. Most γδ T cells directly stimulated by antigens secrete IL-4, which stimulates the growth of B cells and the production of immunoglobulins [79].

NK cells: Research has confirmed that NK cells are the principal producers of IFN-γ. γδ T cells activate NK cells via the 4-1BB-L–4-1BB axis. 4-1BBL is expressed on the surface of γδ T cells and interacts with 4-1BB on the surface of NK cells. Following this interaction, the intracellular domain of 4-1BB recruits TRAF2 (TNFR-associated factor 2) to form a signaling complex. This activation triggers the PI3K/Akt and NF-κB pathways, enabling NK cells to proliferate and prolong their survival, thereby enhancing their cytotoxic effects on target cells. Furthermore, this interaction upregulates the expression of effector molecules in NK cells, such as perforin and granzyme B, further augmenting their killing capacity [80]. Co-stimulatory signals increase the cytotoxicity of NK cells against tumor cells, rendering them more potent.

Dendritic cells (DCs): Vδ2 T cells recruit DCs by releasing the Granulocyte-Macrophage Colony-Stimulating Factor (GM-CSF). DCs of human origin rather than murine monocytes can activate γδ T cells. Moreover, receptor transduction effectively promotes DC maturation. Additionally, γδ T cells induce the expression of CD86 and MHC-I molecules in immature DCs by releasing IFN-γ [81].

### 3.3. The Promoting Effect of γδ T Cells on Tumors

**Figure 4:**
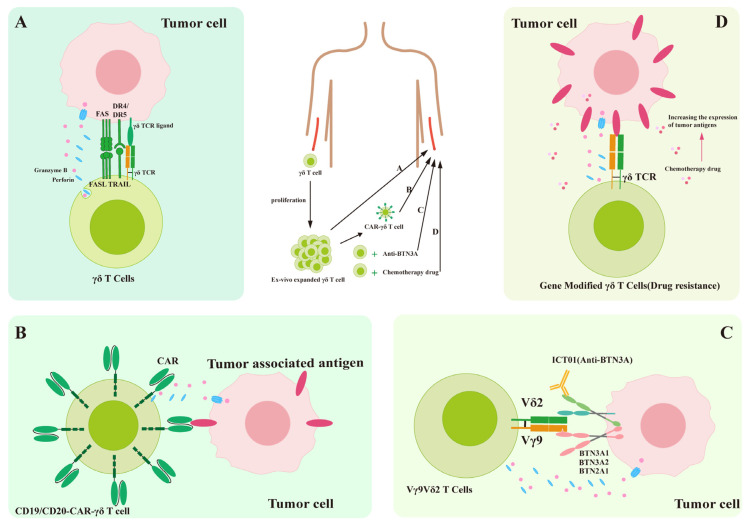
Immunotherapy using γδ T cells. The PBMCs of the patient are extracted from peripheral blood. Following their transformation into potential cancer-fighting γδ T cells, they are administered to the patients as immunotherapy. (**A**) Unmodified adoptive therapy that focuses on harnessing the natural abilities of effector γδ T cells. (**B**) CAR structure embedded into γδ T cells. (**C**) γδ T-cell therapy in combination with immune checkpoint inhibitors. (**D**) γδ T-cell therapy in combination with a chemotherapy drug. This effect enhances the cytotoxic activity of γδ T cells and consequently promotes tumor cell death.

Although γδ T cells in the TME exert outstanding antitumor effects, immunosuppressive cells and associated inhibitory elements can also result in the depletion of γδ T cells, reducing their antitumor effectiveness (Figure 4). Several studies have indicated that tumors formed subcutaneously, in situ, or via the intravenous injection of cancer cell lines in mice increase IL-17A expression by γδ T cells [82]. Wu first discovered that IL-17+ γδ T cells promote the growth of human colorectal cancer [83]. However, an analysis of the functional characteristics of the tumor-infiltrating Vδ1 and Vδ2 T-cell subsets in colorectal cancer and adjacent normal tissues revealed that they mainly secrete IFN-γ, while IL-17 secretion levels were extremely low. It is speculated that these two opposite research results may be related to the different stages of Vδ1 and Vδ2 T cells in tumor tissues. In the microenvironment of human colorectal cancer, the main population secreting IL-17 are innate γδ T cells. This cell subset is activated by IL-23, which is highly expressed in colorectal cancer lesions. The production of IL-23 is mainly attributed to tumor-infiltrating inflammatory dendritic cells [84,85]. IL-17A may directly influence endothelial cells, stimulate tumor growth by enhancing angiogenesis in immunocompetent hosts, or upregulate adhesion molecules and endothelial cell permeability [86]. The suitable conditions provided by the TME for the recruitment of IL-17+ γδ T cells play a vital role in promoting IL-17A expression. In addition to secreting IL-17A to promote tumor growth, γδ T cells also produce IL-4 [87]. In B16 melanoma, IL-4 production indirectly promotes tumor growth by inhibiting the cytotoxicity of additional antitumor γδ T-cell populations.

Studies of colorectal cancer have found a positive correlation between tumor-infiltrating γδT17 cells and IL-17 levels, as well as a link between these cells and tumor progression [84]. IL-17A promotes the proliferation of cancer cells not by directly influencing tumor cell growth but by indirectly facilitating tumor expansion through the modulation of the immune microenvironment. The tumor microenvironment manipulates γδ T cells to favor cancer survival [83]. It converts antitumor γδ T cells into IL-17+ γδ T cells and γδTreg cells. This process involves the inhibition of IFN-γ and granzyme expression while enhancing IL-17 production in γδ T cells. The resultant IL-17 increases VEGF expression, which attracts neutrophils and MDSCs to the tumor site, thereby inhibiting the antitumor activity of CD8+ and CD4+ T cells [88]. IL-17 induces VEGF production through JAK/STAT signaling pathway activation. VEGF serves as a crucial regulator of tumor angiogenesis. It specifically targets VEGF receptors on endothelial cells, promoting their proliferation, migration, and invasion, as well as the formation of new vascular structures [89]. These newly formed blood vessels supply adequate oxygen and nutrients to tumor cells, creating an optimal environment for tumor tissue growth.

### 3.4. Coordination of Additional Cells by γδ T Cells in Promoting Tumors

Neutrophils: Research has demonstrated that IL-1β within the TME stimulates γδ T cells to secrete IL-17A. IL-17A subsequently recruits circulating neutrophils to the tumor site via CXC chemokines produced by epithelial cells, transforming these neutrophils into immunosuppressive cells. These immunosuppressive neutrophils inhibit CD8+ T-cell function and their cytotoxic effect on cancer cells, promoting breast cancer proliferation [90,91]. Additionally, immunosuppressive neutrophils express programmed cell death ligand 1 (PD-L1) on their surface, which interacts with the PD-1 receptor on T cells, suppressing T-cell proliferation and activity. This interaction collaboratively contributes to an immunosuppressive TME, facilitating the progression of hepatocellular carcinoma [92].

MDSCs: During colorectal carcinogenesis, impaired intestinal epithelial barrier function can lead to the translocation of microflora, triggering the recruitment and activation of inflammatory dendritic cells (inf-DCs) in the tumor microenvironment. These inf-DCs then drive the differentiation of γδT17 cell subsets. IL-8, TNF-α and GM-CSF are some of the effector factors released by such polarized γδT17 cells [83]. Both IL-17 and GM-CSF play crucial roles in the mobilization and recruitment of MDSCs in tumor-infiltrating mice [83]. In tumor-bearing mice, IL-17 has been shown to promote the development of MDSCs while inhibiting their apoptosis [93]. Furthermore, in the presence of IL-17, the immunosuppressive activity of MDSCs on T cells is enhanced [94]. PMN-MDSCs preferentially accumulate within the TME compared to M-MDSCs, transforming tumor-induced inflammation into an immunosuppressive milieu [83]. PMN-MDSCs in the TME further inhibit the cytotoxicity of γδ T cells by suppressing IFN-γ secretion from these cells. Concurrently, IL-17A enhances CXCL5 production in tumor cells and promotes MDSC migration at the tumor site through a CXCR2-dependent mechanism, facilitating tumor progression and immune evasion [95].

## 4. Immunotherapy with γδ T Cells

γδ T cells have emerged as highly promising candidates for various immunotherapeutic approaches, owing to their potent and broad antitumor activity, excellent safety profile, and potential for allogeneic use [96] (Table 3). Adoptive therapy with γδ T cells is an immunotherapeutic method that involves expanding and activating the γδ T cells of a patient ex vivo, followed by reinfusion into the patient to enhance immunity and thereby treat the disease [97]. However, further research is needed to determine how to expand and activate these cells most effectively and ensure their sustained and effective functioning. Meanwhile, clinical trials require the establishment of a systematic safety monitoring system.

Despite advancements in immunotherapy, most solid tumors still respond poorly to the existing treatments. However, introducing immune checkpoint inhibitors has significantly changed the treatment of certain tumors [98]. Unfortunately, most human malignancies resist the checkpoint inhibitors that enhance αβ T cell responses [99]. However, γδ T cells are also active in numerous types of human cancers, constituting over 20% of the CD3+ T cells within tumors, where they primarily play a regulatory role. Although γδ T cells can spontaneously exhibit cytotoxicity against tumors, they still require a strong driving force to harness their antitumor activity and expand the scope of immunotherapy (Figure 4) [100].

γδ CAR-T therapy shows unique clinical advantages compared with αβ CAR-T cells. Studies have shown that, due to its inherent limitations (such as genomic instability caused by off-target effects), gene editing technology cannot completely eliminate residual αβ-TCR+ cells from the final product. Even a residual ratio of 1% may induce graft-versus-host disease (GvHD). Furthermore, maintaining the integrity of the endogenous TCR signaling pathway is crucial for the function of CAR-αβ T cells. Its absence leads directly to a shorter survival time of the cells in vivo and decreased tumor-killing efficacy [101,102,103,104]. Notably, γδ CAR-T cells are non-MHC-restricted and therefore fundamentally avoid the risk of GvHD. This feature simultaneously addresses the issue that αβ CAR-T cells cannot recognize MHC-I-negative tumor cells. Vγ9Vδ2 TCR can recognize BTN3A directly or recognize tumor cells via the NKG2D/DNAM-1 axis, a function not possessed by αβ T cells. However, another study shows that CD8+ T cells can kill MHC-I negative tumor cells via the NKG22-NKG2DL axis [105].

**Table 3:** The roles of Vδ1 and Vδ2 T cells in different cancers.

Subset	Tumor Type	Function and Prognostic Association	Role in TME	Potential Therapeutic Strategies	References
Vδ1+ γδ T Cells	Colorectal Cancer	Poor prognosis (MSS type): this comprises 74.4% of γδ T cells with impaired function (reduced levels of cytotoxic molecules such as perforin, granzyme B, and IFN-γ).	Inflammatory fibroblasts overexpress NECTIN2, which binds to TIGIT on Vδ1+ cells, thereby suppressing their activity.	Anti-TIGIT antibodies or NECTIN2 blockade.	[84]
		Favorable prognosis (MSI type): maintains strong cytotoxicity (granzyme B and IFN-γ).	Direct tumor cell killing.	PD-1 inhibitors are effective, but only in a minority of cases.	[84]
	Non-Small Cell Lung Cancer	Favorable prognosis: high abundance of intratumoral Vδ1+ T cells is associated with recurrence-free survival. TCGA data indicate a longer overall survival in patients with high TRDV1 expression.	CD103+ Vδ1+ T cells colonize lung tissue and recognize early tumor stress signals independent of MHC restriction.	Expand CD103+ Vδ1+ T cells ex vivo for adoptive transfer to enhance tumor targeting.	[21]
	Merkel Cell Carcinoma	1. Enriched in MHC-I-deficient tumors, compensating for CD8+ T cell limitations. 2. Vδ1+ clonal expansion correlates with prolonged survival.	1. NKG2D-mediated killing of MHC-I-deficient tumors. 2. Direct recognition of MCPyV viral peptides via TCR.	Design MCPyV peptide vaccines to enhance Vδ1+ T cell expansion.	[106]
	Ovarian Cancer	CD3^+^Vδ1+ T cells are significantly elevated in ovarian cancer patients and correlate with advanced FIGO stage and metastasis.	High Foxp3 and Vδ1 expression, low CD28, maintaining immunosuppressive function and promoting progression.	Target Vδ1+ surface markers (e.g., Vδ1, Foxp3) to block immunosuppression.	[107]
	Hepatocellular Carcinoma (HCC)	1. Increased Vδ1+/Vδ2+ ratio correlates with shorter survival. 2. CD69+ Vδ1+ T cells are antitumor subpopulations linked to smaller tumor size and prolonged survival.	1. Synergizes with apoptosis, ferroptosis, and pyroptosis pathways; PD-1/PD-L1 overexpression. 2. CD69+ Vδ1+ T cells localize to tumor sites for direct cytotoxicity.	1. Combine PD-1/PD-L1 inhibitors to reverse T cell exhaustion. 2. Expand CD69+ Vδ1+ T cells ex vivo for adoptive transfer.	[108,109]
Vδ2+ γδ T Cells	Renal Cell Carcinoma	No direct prognostic correlation, but γδ T cell models (including Vδ2) predict immunotherapy response.	Functionally restricted in high TGF-β or IL-10 environments; requires combination therapy.	Zoledronic acid or BTN3A1 agonists to enhance activity.	[110]
	Colon Adenocarcinoma	Vδ2+ infiltration correlates with inflammation but lacks standalone prognostic value.	Activity depends on tumor BTN3A1 expression and phosphoantigen availability; suppressed in TGF-β-rich TME.	Pre-treat tumor cells with zoledronic acid to increase IPP release and activate Vδ2+ cells.	[111]
	Breast Cancer	Reduced peripheral Vδ2+ T cell levels correlate with tumor progression.	Vγ9Vδ2 TCR recognizes tumor metabolic stress via BTN3A1.	Adoptive transfer of ex vivo-expanded Vδ2+ cells combined with IL-2 to sustain activity.	[112]
	Ovarian Cancer	No significant difference in CD3^+^Vδ2+ T cell proportions between benign and malignant tumors.	Likely not directly involved in immunosuppression.	Not recommended as a therapeutic target.	[107]
	Multiple Myeloma	Reduced peripheral Vδ2+ T cells correlate with advanced disease; bone marrow Vδ2+ T cell infiltration links to relapse/refractory MM.	CXCL10 recruits γδ T cells via CXCR3 into hypoxic bone marrow, promoting IL-17+ polarization.	Restore Vδ2+ function with PD-1 inhibitors combined with SRC-3 inhibitors.	[113]

### 4.1. Research on BTN3A1 and Vγ9Vδ2 T-Cell Immunotherapy

Studies have revealed that the interaction of BTN and BTNL molecules with Vγ9Vδ2 T cells holds potential application value in tumor immunotherapy [114]. Investigating the function of BTN3A1 and its expression in tumor tissues has provided new targets for tumor immunotherapy, allowing researchers to explore novel immune therapeutic strategies to enhance the immune response against tumors [115]. The BTN3A1–BTN2A1 interaction is crucial for stimulating Vγ9Vδ2 T cells dependent on the TCR. In vivo, this relies on the attachment of phosphorylated metabolic products to the B30.2 domain. Nevertheless, these impacts can be replicated by fortifying the external domain of BTN3A1 using CD277-specific antibodies, potentially via the multimerization of BTN3A1 and natural alterations in its V-shaped configuration [116,117,118]. Significant progress has been made in preclinical studies with BTN3A1 recombinant proteins. ICT01, a monoclonal antibody that targets BTN3A, selectively stimulates Vγ9Vδ2 T cells and is presently under investigation in the ongoing phase I/IIa EVICTION trial (NCT04243499) for advanced solid and hematological cancers. ICT01-activated Vγ9Vδ2 T cells can destroy acute myeloid leukemia blasts and lymphoma cell lines in vitro, making Vγ9Vδ2 T cells a promising new immunotherapeutic approach for hematological malignancies [119]. However, given the low proportion of Vγ9Vδ2 T-cell subpopulations, promoting cooperation between two T-cell subpopulations may enhance the benefit of immunotherapy for patients with malignant tumors. In 2020, to block the immunosuppressive activity of BTN3A1, Payne and colleagues screened a series of full-length monoclonal antibodies that react with BTN3A1, ultimately determining that clone CTX-2026 had the best activity. CTX-2026 demonstrated a superior ability to remodel the in vitro activation of CD4+ and CD8+ αβ T cells. Most notably, CTX-2026 activates γδ T cells to eradicate tumor cells while reshaping the antitumor effector function of αβ T cells. The inhibitory effect of BTN3A1 on αβ T cells is seen in its natural form, not necessitating BTN2A1. Antibodies against BTN3A1 can counteract the inhibition of αβ T cells while inducing antitumor cytotoxicity in γδ T cells. Trials have demonstrated that antibodies targeting CD277 can change BTN3A1 from an immune suppressor to an immune stimulator, thereby dynamically eliciting antitumor immunity driven by coordinated αβ T and γδ T cells, halting the progression of ovarian cancer [120]. Treatment with antibodies targeting BTN3A has made progress, while therapy targeting BTN2A1 is still in the early stages. Strategies such as directly targeting and activating BTN2A1 and BTN3A1 to selectively activate Vγ9Vδ2 T cells may become powerful clinical tools [121].

### 4.2. Types of Immunotherapeutic γδ T Cells

Immunotherapies involving γδ T cells can generally be divided into four categories (Table 4). The first is unmodified adoptive therapy using γδ T cells without genetic modification, which focuses on utilizing the natural capabilities of effector γδ T cells and takes advantage of their MHC-independent nature, clinical safety, and ease of production [122]. In order to advance γδ T-cell therapy, it is crucial to develop a standardized process that encompasses expansion, activation and infusion. This will ensure repeatable therapeutic effects and facilitate its widespread adoption. Adoptive therapy with γδ T cells has been applied in the treatment of various diseases: in tumor treatment, it can enhance patient immunity to suppress tumor growth and proliferation; in infectious diseases, it can target pathogens to reduce symptoms; and in autoimmune diseases, it can regulate the function of the immune system to relieve symptoms [123]. However, this method faces limitations. First, a stable source of γδ T cells is essential. The initial design aimed to expand Vγ9Vδ2 T cells in vitro using autologous PBMCs from cancer patients. However, the PBMCs obtained from most cancer patients cannot be effectively expanded and do not meet the requirements for reinfusion. Patients cannot tolerate 100 mL of blood extraction every 2–3 weeks. Consequently, subsequent clinical studies utilized allogeneic cells instead of autologous ones (NCT03183206, NCT03183219). Nevertheless, the clinical safety of allogeneic Vγ9Vδ2 T cell transplantation must be scientifically validated through clinical trials involving patients [122]. To date, no study has reported on allogeneic Vγ9Vδ2 T-cell adoptive therapy specifically addressing severe graft-versus-host disease. Second, it is crucial to optimize the existing expansion methods [96]. Amino bisphosphonates are frequently employed to induce γδ T cell expansion. However, they primarily expand the Vγ9Vδ2 T-cell subset and are ineffective for Vδ1+ T cells. This limitation results in cellular waste and prevents the full utilization of the unique capabilities inherent to Vδ1+ T cells. Methods of expanding Vδ1 T cells have been investigated, and in 2016 the Bruno Silva-Santos team successfully expanded Vδ1 T cells (without genetic modification, 70 ng/mL rIL-15, 30 ng/mL IFN-γ, and 1 μg/mL anti-CD3 mAb)) expressing NKp30 and NKG2D on the cell surface, known as Delta One T cells (DOT cells), for the treatment of hematological malignancies [124]. In 2025, the team will enhance the cytotoxicity of DOT cells by using butyrate to upregulate NKG2D expression to improve the targeting of tumor cells, and block both PD-1 and TIGIT to treat colorectal cancer [125]. Third, incorrect activation may occur, partly due to helper T-cell deficiency [6,126]. The polyclonal γδ T-cell reserves received by patients lead to insufficient concentrations of tumor-reactive γδ T cells. Fourth, there are potential off-target effects. The recognition of tumor cells by Vγ9Vδ2 T cells necessitates PAg binding within tumor cells through the B30.2 domain of BTN2A1–BTN3A1. This may lead to compromised Vγ9Vδ2 T-cell functionality [15].

The second category is modified adoptive therapy, in which the classic CAR structure is embedded into γδ T cells as the starting point. The targets of CAR-γδ T cells can be divided into two groups: antigens that are highly expressed in tumors and receptors, such as NKG2DL and PD-L1. Modified γδ T cells can target and kill tumor cells more effectively, thereby improving the specificity and efficacy of treatment. The advantage of CAR-γδ T-cell therapy is its broad antitumor activity, attacking various types of tumors without needing to pre-identify specific antigens [127]. One direction for improvement is to optimize the CAR molecular design using high-affinity single-chain antibody fragments as the recognition domain and introducing additional co-stimulatory signal domains to enhance the function of CAR-γδ T cells. Moreover, modifications in CAR design can potentially alleviate the depletion of engineered γδ T cells, a longstanding issue that has significantly impacted the clinical efficacy of CAR T-cell therapies. Depletion remains a critical challenge that adversely affects the effectiveness of all T-cell treatments. However, in the context of CAR-γδ T-cell therapy, remission can be attained through structural alterations to the CAR framework [128]. The Cooper team achieved large-scale production using the Sleeping Beauty transposition subsystem. By combining artificial antigen-presenting cells (aAPCs) and induced pluripotent stem cell (iPSC) technology, they expanded CD19 CAR γδ T cells to a quantity suitable for clinical use (10^9^), paving the way for multiple infusions [129]. To optimize safety and specificity, the Schaft team used mRNA electroporation to instantaneously express Melanoma-Associated Chondroitin Sulfate Proteoglycan-specific CAR, achieving a 40% lysis rate in melanoma without the risk of genomic integration [130]. The Anderson team designed the GD2-DAP10 costimulatory receptor, which enhances targeting by relying on endogenous TCR signals to kill tumor cells precisely while preserving healthy tissue in glioblastoma models [131]. The team of Tong Aiping has developed a CD5-CAR-γδT-cell therapy based on mRNA engineering to treat T-ALL [132]. mRNA technology is safe and cost-effective. The CD5 gene of γδT cells was knocked out using CRISPR/Cas9 to create anti-fratricidal CD5-CAR-γδTCD5 cells, which target T-ALL cells using the high-affinity nanobody CD5-27-NB. These cells exhibit a potent tumor-killing effect in both in vitro and in vivo settings, without significant toxicity in normal tissue. The tumor microenvironment, which is inhibited by the PD-1/PD-L1 axis, prevents γδ T cells from exerting their normal cytotoxic effects and killing tumor cells. γδ T cells can be genetically engineered to simultaneously target tumor antigens and block the PD-1 pathway [133]. Introducing membrane-bound anti-PD-1 antibodies into expanded Vγ9Vδ2 T cells to create “armored” γδ T cells significantly improves the treatment of ovarian tumors in mice, providing a basis for clinical trials of combined γδ T cell and PD-1 treatments. The clinical trials in this area are ongoing [134]. The limitations including the following: Studies have shown that PD-1 regulates TCR-activated Vγ4 γδ T cells; however, γδ T cells produced by cytokine-activated IL-17A escape the regulatory effect of the PD-1-PD-L1 pathway. It is possible that PD-1 antibodies are unable to activate all types of γδ T cell [135]. This strategy also faces limitations. First, the DAP10 signal, which NKG2D typically provides, plays a crucial role in activating γδ T cells [136]. However, tumor cells may obstruct this signaling pathway by downregulating or shedding NKG2D ligands, thereby facilitating tumor immune evasion. Tumor cells employ intricate and varied signaling pathways and survival mechanisms [137]. Consequently, targeting only a single molecule may prove insufficient for effectively inhibiting tumor growth and metastasis. Second, CAR-γδ T-cell therapy has been associated with potential side effects such as fever, chills, and headaches. Therefore, thoroughly assessing the condition and physical status of the patient when administering CAR-γδ T-cell therapy is imperative. The close monitoring of therapeutic efficacy and adverse effects is also essential [128]. Nevertheless, the promise of CAR-γδ T-cell therapy should not be overlooked. Future advancements in this therapeutic approach warrant continued attention.

The third category is an antibody-based combination γδ T-cell adoptive therapy. Firstly, using anti-BTN antibodies to stimulate Vγ9Vδ2 T cells, early phase I/II clinical trials revealed a 36% disease control rate in a group of 22 patients treated with ICT01 (anti-BTN3A antibody) [8]. The advantages of this treatment are its strong targeting ability, as the anti-BTN3A antibody can specifically recognize BTN3A molecules on the surface of tumor cells, avoiding damage to normal cells; provision of high safety; and independence from chemotherapy drugs or radiotherapy, reducing the treatment burden and side effects for the patient. In clinical trials, the anti-BTN3A antibody combined with γδ T-cell therapy has shown promising therapeutic effects. However, this treatment method is still in the research stage and requires further investigation. A limitation is that an intravenous injection of the antibody may result in its binding to BTN3A in healthy cells, since most healthy cells also express BTN3A. This binding may alter the conformation of BTN3A and trigger γδ T-cell activation in healthy tissues, potentially leading to adverse side effects [138]. However, no adverse side effects were observed in the experiments involving non-human primates. Clinical trials are currently underway to investigate anti-BTN3A antibodies further. ICT01 is designed to mitigate this issue as an FC-silent IgG1, with no cytotoxicity to normal tissues. However, the specific underlying mechanism remains unexplained [8].

The fourth category combines chemotherapeutic drugs with γδ T cells (NCT04165941). Chemotherapy escalates the presentation of tumor-associated antigens (TAAs) on cancer cells, strengthening the interaction between CAR-γδ T cells and cancer cells. When chemotherapy drugs attack cancer cells, they not only directly kill some of the cancer cells but also change the permeability of the cell membrane, leading to TAA exposure within cells. Under normal circumstances, these antigens are usually unrecognized or inaccessible to the immune system. However, once chemotherapy drugs release them onto the cell surface, T cells and other immune cells can more easily recognize and attack these specific antigen-bearing cancer cells. The advantages of this treatment include enhanced immune response. Chemotherapy can also reduce the tumor burden, thereby alleviating immune suppression, promoting γδ T-cell function, and reducing drug resistance. Combining chemotherapy drugs and immunotherapy can reduce tumor cell resistance to single treatments. One limitation is that the side effects of chemotherapy may overlap with those of immunotherapy, requiring careful management. Ongoing clinical trials are exploring the optimal combination of chemotherapy and γδ T-cell adoptive therapy for different types and stages of cancer, with future research focusing on optimizing treatment plans to maximize efficacy and minimize side effects.

**Table 4:** Ongoing and past clinical trials using γδ T cells.

Clinical Trials Gov Identifier	Interventions	Cancers/Tumors	Phase	Outcomes/Preliminary Findings
**Autologous/Allogeneic γδ T cells**				
NCT02418481	γδ T cells with or without DC-CIK cells	Breast Cancer	I/II	No published results (Study Completion June 2016).
NCT02425735	Vγ9Vδ2 T cells with or without DC-CIK cells	Cholangiocarcinoma	I/II	Modulated immune functions, reduced tumor activity, enhanced quality of life, and extended lifespan. Following eight γδ T cell treatments, there was a significant reduction in lymph node size along with diminished activity [123].
NCT02425748	γδ T cells with or without DC-CIK cells	Lung Cancer	I/II	No published results (Study Completion 20 June 2019). Offer another promising immunotherapy approach.
NCT02585908	Vγ9Vδ2 T cells with or without CIK cells	Gastric Cancer	I/II	No published results (Study Completion December 2022).
NCT03180437	Vγ9Vδ2 T cells with IRE surgery	Locally Advanced Pancreatic Cancer	I/II	Strengthened immune response, inhibited tumor expansion, and prolonged the survival of liver and pancreas cancer patients [139].
NCT03183232	γδ T cells with Cryosurgery or IRE	Liver Cancer Lung Cancer	I/II	Decreased tumor volume and increased survival in mice. Allogeneic Vγ9Vδ2 T cells have shown clinical safety and initial evidence of therapeutic effectiveness in patients with solid tumors [122].
NCT03533816	Ex-vivo Expanded/Activated γδ T-cell Infusion	Hematological Malignancies	I	Assessing the maximum tolerated dose and safety profile of autologous gamma-delta T cells in leukemia patients who have undergone a partially matched bone marrow transplant.
NCT03790072	Ex-vivo Expanded Allogeneic γδ T-lymphocytes (OmnImmune^®^)	Acute Myeloid Leukemia	I	Allogeneic Vγ9Vδ2 T-cell infusion was shown to be safe and feasible up to a cell dose of 10^8^/kg [140].
NCT04764513	Ex-vivo expanded γδ T-cell infusion	Acute Myeloid Leukemia Acute Lymphoblastic Leukemia Myelodysplastic Syndromes Lymphoma	I/II	Recruiting (Study Completion December 2025).
NCT04518774	Ex-vivo expanded Allogeneic γδ T cells	Hepatocellular Carcinoma	Early Phase I	No published results (Study Completion 15 August 2021).
NCT04696705	Ex-vivo expanded Allogeneic γδ T cells	Non-Hodgkin’s Lymphoma and Peripheral T-Cell Lymphomas	Early phase I	No published results (Study Completion 25 December 2023).
NCT04765462	Allogeneic γδ T cells	Malignant Solid Tumors	I/II	No published results (Study Completion 31 December 2024).
NCT05015426	γδ T-Cell Infusion	Acute Myeloid Leukemia	I	Not Recruiting (Study Completion September 2026).
NCT05358808	Ex-Vivo expanded Allogeneic γδ T-lymphocytes (TCB008)	Acute Myeloid Leukemia Myelodysplastic Syndromes	II	Recruiting (Study Completion December 2025).
NCT05628545	Allogeneic γδ-T Cells (GDKM-100)	Advanced Hepatocellular Carcinoma	I/II	No published results (Study Completion 31 October 2024).
NCT05886491	Allogeneic Vδ1 T cells	Acute Myeloid Leukemia	I/II	Recruiting (Study Completion 30 June 2027).
**CAR-γδ T cell**				
NCT02656147	Anti-CD19-CAR-γδ T cell	Leukemia and Lymphoma	I	No published results (Study Completion April 2020).
NCT04107142	NKG2DL-targeting CAR-γδ T cell	Solid Cancer	I	NKG2DL-targeting CAR-γδ T cells enhanced cytotoxicity against tumor cell lines, with Vγ9Vδ2 T cells modified by NKG2D RNA-based CAR showing notable therapeutic effects in mouse tumor models [141].
NCT04702841	CAR-γδ T cell	Relapsed and Refractory CD7 positive T	I	No published results (Study Completion December 2022).
NCT04735471/ NCT04911478/ NCT06375993	ADI-001 Anti-CD20 CAR-engineered Allogeneic γδ T Cells	Lymphoma, Follicular Lymphoma, Mantle-Cell Marginal Zone Lymphoma Primary Mediastinal B-cell Lymphoma/Lupus Nephritis Autoimmune Diseases	I	CD20 CAR-modified Vδ1 γδ T cells did not cause xenogeneic graft-versus-host disease in immunodeficient mice. They demonstrated tumor cell lysis in vitro and proinflammatory cytokine release, as well as inhibition of B-cell lymphoma xenograft growth in immunodeficient mice [142].
NCT05388305	CAR-γδ T cell	Acute myeloid leukemia	Not applicable	No published results (Study Completion 30 May 2023).
NCT05302037	Allogeneic NKG2DL-targeting CAR-grafted γδ T cells (CTM-N2D)	Malignancy Refractory Cancer	I	Recruiting (Study Completion December 2026).
NCT05554939	Allogeneic CD19-CAR-γδ T cell	Non-Hodgkin’s Lymphoma	I/II	Recruiting (Study Completion 31 December 2026).
NCT05653271	Allogeneic CD20-conjugated γδ T-cell	B-cell Lymphoma Non-Hodgkin’s Lymphoma Primary Mediastinal Large B Cell Lymphoma	I	Recruiting (Study Completion September 2027).
NCT06106893	CD19 Universal CAR-γδ T cells	Systemic Lupus Erythematosus	I/II	Recruiting (Study Completion December 2027).
NCT06150885	CAR-γδ T cells CAR001	Solid Tumor	I/II	Recruiting (Study Completion 30 September 2027).
NCT06404281	γδ T-PD-1 Ab cells	Advanced Solid Tumors	I	Recruiting (Study Completion 1 June 2026).
NCT06480565	ADI-270 (engineered γδ Chimeric Receptor CAR Vδ1 T cells Targeting CD70)	Clear Cell Renal Cell Carcinoma	I/II	Recruiting (Study Completion June 2027).
**Antibodies with Autologous/Allogeneic γδ T cells**				
NCT04243499	Anti-BTN3A	Hematological and Solid Ttumors	I/II	Good tolerability and pharmacodynamic activity in initial patients, with the potential to enhance immune cell infiltration into the tumor microenvironment [8].
NCT06364800	Allogeneic γδ T cells combined with targeted therapy and PD-1	Hepatocellular Carcinoma	Early Phase 1	Recruiting (Study Completion 26 September 2026).
NCT06212388	Allogeneic γδ T cells Combined with Interferon-alpha1b or PD-1	Melanoma	Early Phase 1	Recruiting (Study Completion 30 October 2028).
**Drug with Autologous/Allogeneic γδ T cells**				
NCT04165941	Drug Resistant Immunotherapy with Activated, Gene-Modified γδ T cells	Glioblastoma Multiforme	I	Increased median survival in mice [143].
NCT05400603	Ex Vivo Expanded Allogeneic γδ T cells in Combination with Dinutuximab, Temozolomide, Irinotecan, and Zoledronate	Neuroblastoma Refractory Neuroblastoma Relapsed Neuroblastoma Relapsed Osteosarcoma Refractory Osteosarcoma	I	Recruiting (Study Completion December 2025).
NCT05664243	Gene-Modified Allogeneic or Autologous γδ T cells	Glioblastoma	I/II	Recruiting (Study Completion December 2025).
NCT06364787	Allogeneic Gamma-delta T cells combined with targeted therapy and immunotherapy	Hepatocellular Carcinoma	I	Recruiting (Study Completion September 2026).

## 5. Conclusions

Understanding the function of γδ T cells in targeting tumor cells is crucial for both basic and clinical research. Our knowledge of γδ T cells is rooted in over 20 years of extensive research, highlighting their unique ability to directly recognize tumor cell antigens and their biological advantages over cytotoxic T lymphocytes [139]. γδ T cells act independently of MHC molecules for antigen recognition, allowing them to circumvent the common immune evasion mechanisms employed by tumor cells, thus demonstrating significant antitumor potential. In this review, we offer an exhaustive overview of the classification of human γδ T cells, the structure of γδ T cell recognition by tumor cells, and the function of γδ T cells in targeting tumor cells, revealing their unique role in the TME. Vγ9Vδ2 T cells identify tumors in a specific manner distinct from other immune cells. This process involves abnormal energy metabolism in tumor cells, leading to the expression of BTN family genes and the production of PAgs via MVP. The accumulated PAgs associate with B30.2 of BTN3A1, forming complexes including BTN2A1 and BTN3A2. This intracellular binding causes conformational changes in the extracellular domains of BTN molecules, transmitting signals from within the tumor cell externally, which are then identified by Vγ9Vδ2 T cells [144].

Despite these advancements, our understanding of γδ T cell-focused tumor cell targeting and removal is still in the early stages. Research on γδ T-cell immunotherapy represents a highly forward-looking topic within the medical field, and its significance in future clinical applications cannot be overstated. Although the processes of PAg-induced Vγ9Vδ2 T cell stimulation are well studied, further research is needed to uncover the connections and interactions of both PAg and Vγ9Vδ2 T cells with BTN family molecules. The molecular mechanisms and participants involved in this process remain largely unknown. Moreover, investigating the integration of Vγ9Vδ2 T cells with tumor cell surface molecules may reveal potential targets for tumor immunotherapy to develop more efficient, safe, and targeted immunotherapy methods [145]. In summary, in-depth research on the cellular processes by which Vγ9Vδ2 T cells identify tumor cells at the molecular level is crucial for advancing adoptive therapy and immune checkpoint-based tumor immunotherapy using Vγ9Vδ2 T cells [146]. This research will advance the medical field and significantly contribute to human health.

## Data Availability

The data used in this review are derived from public resources, including but not limited to well-known academic databases, publicly available government reports, and authoritative websites in specialized fields. The acquisition of all data complies with the relevant laws and regulations, as well as the terms of use stipulated by the data providers. For the data obtained from academic databases, their sources have been detailed in the references, and readers can trace the original data based on the cited information. Data from government reports can be accessed through the official websites of the respective government departments. Data from authoritative websites in specialized fields are provided with links or clear source descriptions at appropriate locations in the text. The purpose of this statement is to ensure the transparency and reproducibility of the data, thereby promoting the healthy development of academic research. If you have any special requirements regarding the sources and acquisition methods of the data in this report, or if you would like to provide additional information, please feel free to let me know.

## Abbreviations

The following abbreviations are used in this manuscript:

PAg	Phosphoantigen
MVP	Mevalonate pathway
BTN3A1	Butyrophilin Subfamily 3 Member A1
BTN2A1	Butyrophilin Subfamily 2 Member A1
TME	Tumor microenvironment
MHC	Major histocompatibility complex
αβT	Alpha-Beta T
γδ T	Gamma-Delta T
TCR	T Cell Receptor
VEGF	Vascular endothelial growth factor
GM-CSF	Granulocyte-Macrophage Colony-Stimulating Factor
IPP	Isopentenyl Pyrophosphate
APCs	Antigen-Presenting Cells
DMAPP	Dimethylallyl Pyrophosphate
HMBPP	Hydroxymethylbutenyl Diphosphate
HMGR	3-Hydroxy-3-Methylglutaryl-CoA Reductase
MEP	methylerythritol phosphate pathway
β2M	β2-microglobulin
JM	juxtamembrane
NK cell	Natural killer cell
DC	Dendritic cell
TAAs	Tumor-associated antigens
